# RETRACTED: Ali et al. Blockchain-Powered Healthcare Systems: Enhancing Scalability and Security with Hybrid Deep Learning. *Sensors* 2023, *23,* 7740

**DOI:** 10.3390/s26144609

**Published:** 2026-07-21

**Authors:** Aitizaz Ali, Hashim Ali, Aamir Saeed, Aftab Ahmed Khan, Ting Tin Tin, Muhammad Assam, Yazeed Yasin Ghadi, Heba G. Mohamed

**Affiliations:** 1 School of IT, UNITAR International University, Petaling Jaya 47301, Malaysia; 2 Department of Computer System, Abdul Wali Khan University Mardan (AWKUM), Mardan 23200, Pakistan; 3 Department of Computer Science and IT, Jalozai Campus, UET Peshawar, Peshawar 25000, Pakistan; 4 Department of Computer Science, Abdul Wali Khan University Mardan (AWKUM), Mardan 23200, Pakistan; 5 Faculty of Data Science and Information Technology, INTI International University, Nilai 71800, Malaysia; 6 Department of Software Engineering, University of Science and Technology Bannu, Bannu 28100, Pakistan; 7 Department of Computer Science and Software Engineering, Al Ain University, Abu Dhabi 122612, United Arab Emirates; 8 Department of Electrical Engineering, College of Engineering, Princess Nourah bint Abdulrahman University, P.O. Box 84428, Riyadh 11671, Saudi Arabia

The journal retracts the article titled “Blockchain-Powered Healthcare Systems: Enhancing Scalability and Security with Hybrid Deep Learning” [1], cited above.

Following publication, concerns were brought to the attention of the Editorial Office regarding the integrity and originality of data published in [1].

In accordance with standard journal procedures, the Editorial Office and Editorial Board conducted an investigation that identified indications of dataset misrepresentation and image duplication between this article [1] and an early publication [2], produced by a different author and presenting different experimental conditions. The investigation also identified an inappropriate level of self-citation. Although the authors cooperated with the investigation, the explanations and supporting materials provided were not deemed sufficient by the Editorial Board to resolve the identified concerns. As a consequence, the Editorial Board has lost confidence in the reliability of the reported findings and has decided to retract this publication in accordance with MDPI’s retraction policy.

This retraction was approved by the Editor-in-Chief of the *Sensors* journal.

The authors have been informed of this retraction and have indicated their disagreement with this decision.
